# Dose-dependent decrease in anti-oxidant capacity of whole blood after irradiation: A novel potential marker for biodosimetry

**DOI:** 10.1038/s41598-018-25650-y

**Published:** 2018-05-09

**Authors:** Lue Sun, Yohei Inaba, Keizo Sato, Aki Hirayama, Koji Tsuboi, Ryuji Okazaki, Koichi Chida, Takashi Moritake

**Affiliations:** 1 Department of Radiation Biology, Faculty of Medicine, University of Tsukuba, 1-1-1 Tennodai, Tsukuba, Ibaraki 305-0006 Japan; 2 Department of Radiological Health Science, Institute of Industrial Ecological Sciences, University of Occupational and Environmental Health, Japan, 1-1 Iseigaoka, Yahatanishi-ku, Kitakyushu, Fukuoka 807-8555 Japan; 3 Course of Radiological Technology, Health Sciences, Tohoku University Graduate School of Medicine, 2-1 Seiryo, Aoba, Sendai, Miyagi 980-8575 Japan; 4 Department of Radiation Disaster Medicine, International Research Institute of Disaster Science, Tohoku University, Aramaki Aza-Aoba 468-1, Aoba-ku, Sendai 980-0845 Japan; 5 School of Pharmacy, Kyushu University of Health and Welfare, 1714-1 Yoshino Nobeoka, Miyazaki, 882-8508 Japan; 6 Center for Integrative Medicine, Tsukuba University of Technology, Kasuga 4-12-7, Tsukuba, 305-8521 Japan

## Abstract

Many reports have demonstrated that radiation stimulates reactive oxygen species (ROS) production by mitochondria for a few hours to a few days after irradiation. However, these studies were performed using cell lines, and there is a lack of information about redox homeostasis in irradiated animals and humans. Blood redox homeostasis reflects the body condition well and can be used as a diagnostic marker. However, most redox homeostasis studies have focused on plasma or serum, and the anti-oxidant capacity of whole blood has scarcely been investigated. Here, we report changes in the anti-oxidant capacity of whole blood after X-ray irradiation using C57BL/6 J mice. Whole-blood anti-oxidant capacity was measured by electron spin resonance (ESR) spin trapping using a novel spin-trapping agent, 2-diphenylphosphinoyl-2-methyl-3,4-dihydro-2H-pyrrole N-oxide (DPhPMPO). We found that whole-blood anti-oxidant capacity decreased in a dose-dependent manner (correlation factor, r > 0.9; P < 0.05) from 2 to 24 days after irradiation with 0.5–3 Gy. We further found that the red blood cell (RBC) glutathione level decreased and lipid peroxidation level increased in a dose-dependent manner from 2 to 6 days after irradiation. These findings suggest that blood redox state may be a useful biomarker for estimating exposure doses during nuclear and/or radiation accidents.

## Introduction

Biodosimetry is essential for estimating ionizing radiation exposure after large-scale radiological and/or nuclear incidents. It is also used to assign people with significant exposure to appropriate medical care, ideally within the first 2–3 days^1^. In addition, biodosimetry has an important role in long-term studies of radiation health risks^2^. Several biomarkers responsive to radiation have been identified. Examination of chromosome aberration frequencies in lymphocytes by fluorescence *in situ* hybridization and the measurement of radicals in tooth enamel using electron spin resonance (ESR) are considered the most suitable biodosimetry techniques^2^. However, combined biodosimetry techniques are appropriate from the standpoint of eliminating individual differences and increasing reliability.

Radiation damages DNA through the direct ionization of DNA components or the ionization of H_2_O to generate reactive oxygen species (ROS) that subsequently oxidize DNA components. In addition, radiation gives rise to delayed cellular oxidative stress a few hours to a few days after irradiation through stimulation of mitochondrial ROS production. Radiation-induced delayed oxidative stress partly contributes to apoptosis, cell death, and mutations^3–5^. However, only a few studies have investigated whether radiation affects the *in vivo* redox state, including ROS, oxidant, and anti-oxidant levels.

Redox balance is very important for aerobic organisms. At suitable levels, ROS function in physiological cell processes. However, increased ROS quantities and/or the decreased efficacy of anti-oxidant systems lead to oxidative stress, which has been implicated in many pathological conditions^6^. Therefore, oxidant and anti-oxidant levels reflect individual differences in health, disease, diet, and lifestyle. Although half of blood volume comprises cellular components (mainly red blood cells (RBCs)) and the other half comprises non-cellular components (plasma and serum), almost all studies have focused on only non-cellular components^7,8^. Recently, Chaleckis *et al*.^9^ evaluated young and elderly human RBCs for plasma metabolites by liquid chromatography-mass spectrometry and demonstrated that 6 of 14 age-related metabolites were enriched in RBCs, including the redox-related metabolites oxidized glutathione (GSSG), NAD^+^, and NADP^+^. These findings suggested that RBC redox state is an important marker for health and body condition.

In the present study, we sought to determine the changes in anti-oxidant capacity following irradiation *in vivo* using a novel ESR spin-trapping technique termed i-STrap (Fig. S1a–c). Furthermore, we investigated whether radiation induced changes in RBC glutathione level, plasma hydroperoxide level, and plasma anti-oxidant capacity. We found that whole-blood anti-oxidant capacity decreased in a dose-dependent manner. RBC glutathione and plasma hydroperoxide levels were partly altered in association with whole-blood anti-oxidant capacity. These findings suggest that redox-related markers may be useful biomarkers for estimating exposure doses during nuclear and/or radiation accidents. Moreover, the low anti-oxidant capacity persisted for at least 50 days after irradiation with ≥2 Gy, suggesting that it may contribute to the pathogenesis of radiation-related late effects, such as carcinogenesis, cataracts, and arteriosclerosis, which are closely related to oxidative stress^10–12^.

## Results

### Evaluation of the characteristics of i-STrap

First, we evaluated the characteristics of i-STrap. As shown in Fig. S2a, the signal intensity increased as the volume of 2-diphenylphosphinoyl-2-methyl-3,4-dihydro-2H-pyrrole N-oxide (DPhPMPO) increased. In contrast, the signal intensity decreased as the blood/saline ratio increased (Fig. S2b). These results demonstrated that i-STrap is based on competitive reactions of anti-oxidants in blood or DPhPMPO with tert-butyl hydroperoxide (tBuOOH)-induced radicals (mainly tBuOO•)^13^ (Fig. S1c). Next, we evaluated the correlations between signal intensity and the number of white blood cells (WBCs) and RBCs in twelve mice and found that the signal intensity decreased as the number of RBCs increased (Fig. S2c). However, we did not find a correlation between signal intensity and the number of WBCs (Fig. S2c). In addition, we mixed blood samples from two mice and centrifuged this sample to separate plasma and RBCs. We then remixed the plasma and RBCs at arbitrary ratios to create 12 remixed blood samples with different numbers of RBCs (Fig. S2d). We performed i-STrap and found that the signal intensity decreased as the number of RBCs increased (Fig. 2d). These results suggested that RBCs or their constituents strongly affect the signal intensity.

We examined whether treatment with three anti-oxidants, ascorbic acid, N-acetyl-L-cysteine (NAC), and the vitamin E analogue Trolox, affected the results of i-STrap. The anti-oxidants were dissolved in saline or dimethyl sulfoxide (DMSO) and mixed with blood samples. We found that the three anti-oxidants did not affect the signal intensity within their normal ranges in mouse plasma (ascorbic acid, 40–60 μM^14^; cysteine, 20–30 μM^15^; and vitamin E, 6–8 μM)^16^ (Fig. S3). These data support the interpretation that RBCs or their constituents strongly affect the signal intensity of i-STrap, while non-cellular components of blood (plasma or serum) have little effect on the signal intensity.

### Dose-dependent decrease in whole-blood anti-oxidant capacity after irradiation

**Figure 1:**
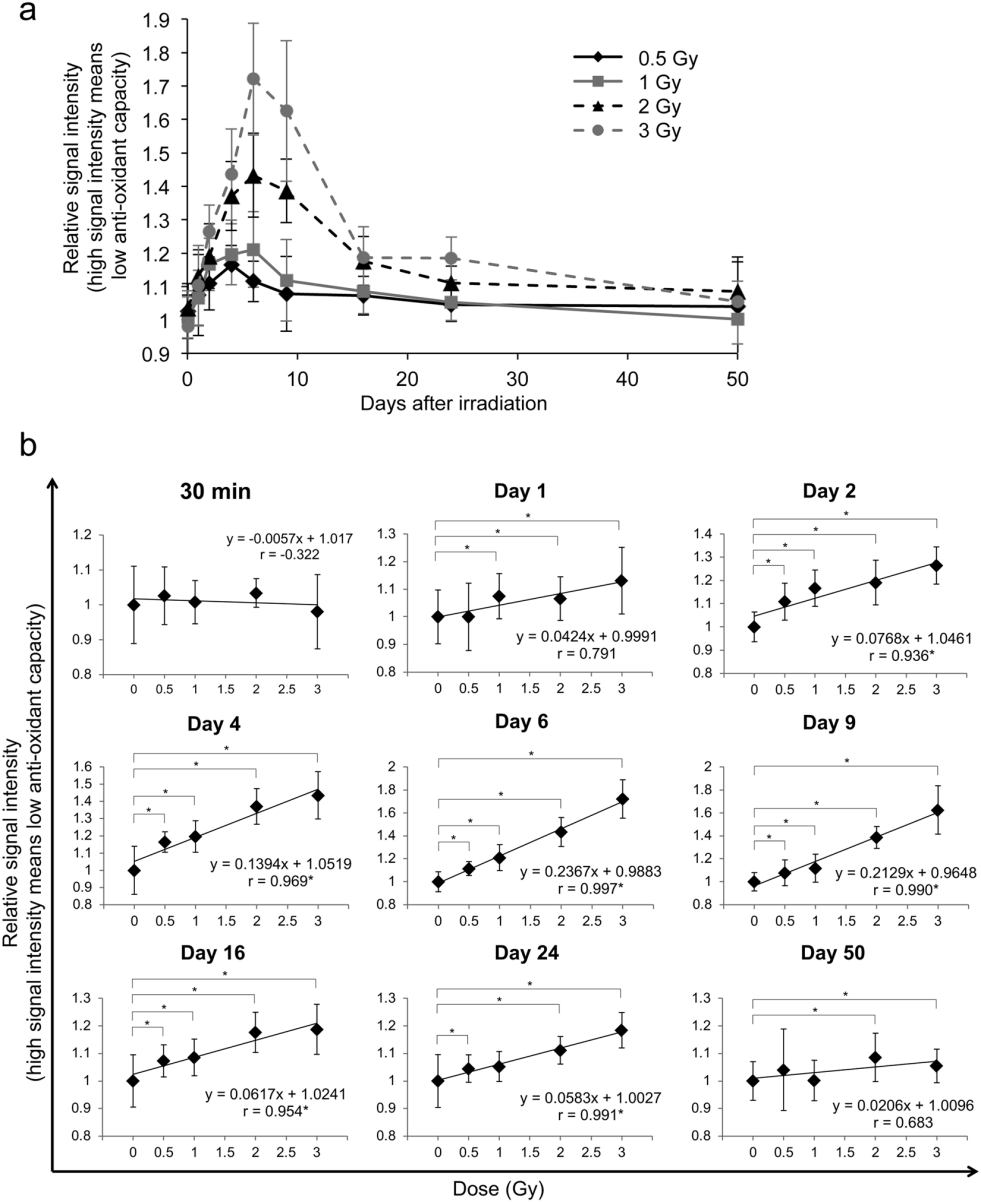
Changes in whole-blood anti-oxidant capacity after irradiation. Whole-blood anti-oxidant capacity was measured using i-STrap. High signal intensity means low anti-oxidant capacity. (**a**) Changes in anti-oxidant capacity after irradiation. (**b**) Evaluation of dose dependence at various time points. All quantitative data are presented as the means ± SD (n = 12–14). *P < 0.05, Welch’s *t*-test and Pearson’s correlation coefficient test.

We examined the anti-oxidant capacity of whole blood from 30 min to 50 days after irradiation at 0.5–3 Gy using i-STrap (Fig. S1). We found that the 0.5 and 1 Gy irradiation groups decreased in anti-oxidant capacity from immediately after irradiation to day 2, retained this lower anti-oxidant capacity until day 6 and then recovered their anti-oxidant capacity to the control (non-irradiated) level by day 24 (Fig. 1a). The 2 and 3 Gy irradiation groups showed decreased anti-oxidant capacity until day 6 and then started to recover (Fig. 1a). However, the anti-oxidant capacity in these two groups did not recover to the control (non-irradiated) level until day 50 (Fig. 1a,b). The whole-blood anti-oxidant capacity decrease showed a significant and very high correlation with irradiation dose (correlation factor, r > 0.9; P < 0.05) 2 to 24 days after irradiation, suggesting its potential for use as a novel biodosimetry marker (Fig. 1b).

### Decreased glutathione levels in RBCs after irradiation

**Figure 2:**
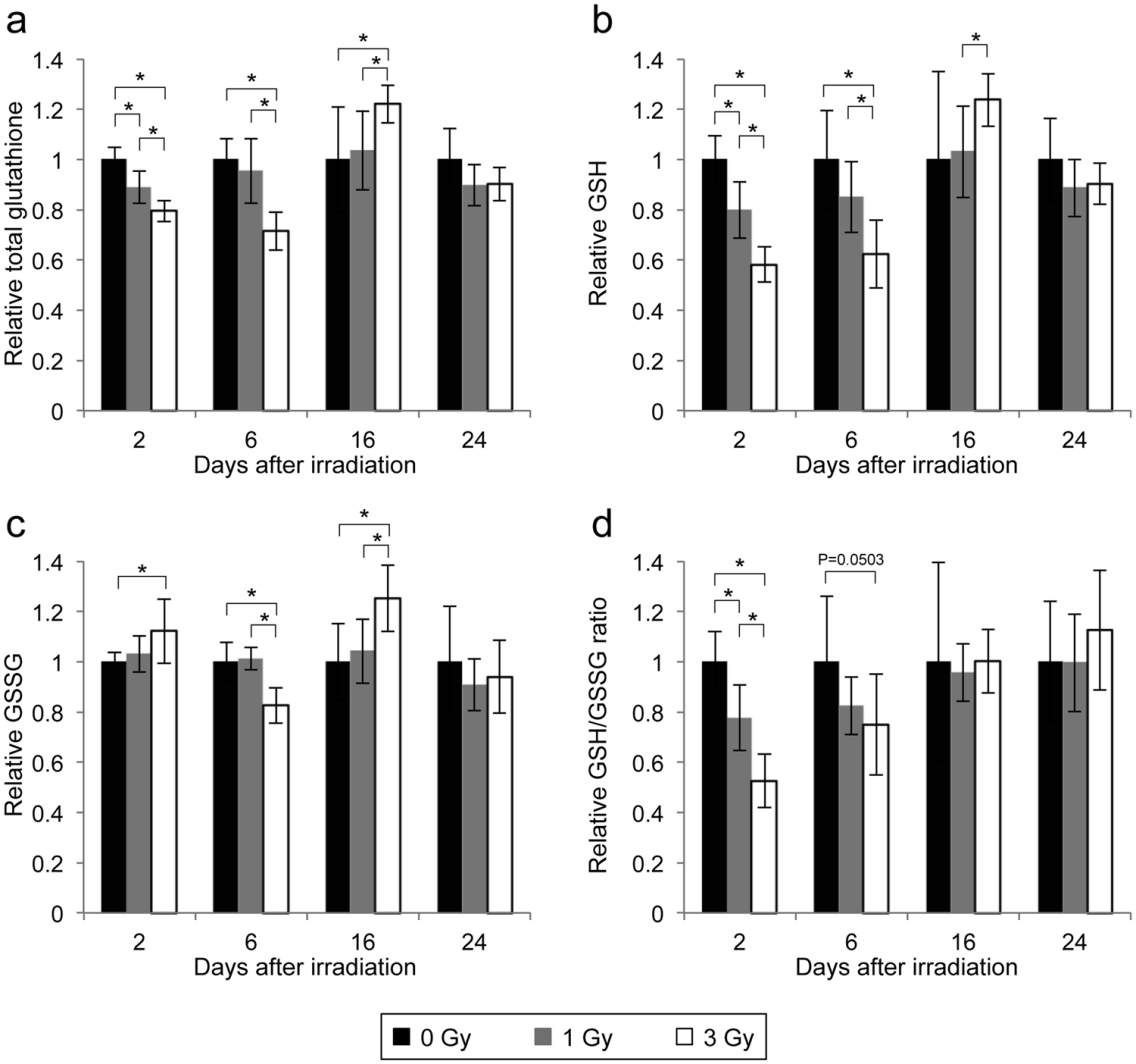
Changes in RBC glutathione levels after irradiation. (**a**–**d**) Changes in RBC total glutathione (**a**), GSH (**b**), and GSSG (**c**) and the GSH/GSSG ratio (**d**) after irradiation. All quantitative data are presented as the means ± SD (n = 6–8). *P < 0.05, Welch’s *t*-test.

Reduced glutathione (GSH) is the most abundant low-molecular-weight thiol and the principal thiol redox buffer in RBCs^17^. Therefore, we explored the changes in total glutathione, GSH, GSSG, and the GSH/GSSG ratio in RBCs after irradiation. At 2 days after irradiation, the levels of total glutathione and GSH and the GSH/GSSG ratio showed dose-dependent decreases, and the 3 Gy irradiation group had a significantly higher level of GSSG than the no-irradiation group (Fig. 2). At day 6, the level of GSH showed a dose-dependent decrease, and the 3 Gy irradiation group had significantly lower levels of total glutathione and GSSG than the no-irradiation group (Fig. 2). At day 16, in contrast to days 2 and 6, the 3 Gy irradiation group had higher levels of total glutathione, GSH, and GSSG than the no-irradiation group (Fig. 2). At day 24, all of the parameters had returned to their levels in the no-irradiation group (Fig. 2).

To investigate whether glutathione decline directly affected the i-STrap results, we inhibited glutathione biosynthesis using buthionine sulfoximine (BSO)^18^. BSO specifically and irreversibly inhibits the rate-limiting enzyme in GSH synthesis, γ-glutamyl-cysteine synthetase (γ-GCS)^19^. We added 10 mM BSO to the drinking water that was administered to the mice for 7 days. BSO treatment induced decreases of approximately 30% in total glutathione and 40% in GSH (Fig. S4a) without any serious side effects (e.g., death, losing weight, and behavioural abnormalities; data not shown). These glutathione levels were almost the same as the levels observed at 2 days after irradiation with 3 Gy. However, BSO treatment did not affect the i-STrap results, suggesting that RBC glutathione did not directly affect the results of i-STrap (Fig. S4b). Nevertheless, the levels of total glutathione and GSH in RBCs and the i-STrap results showed similar changes after irradiation. These two parameters may thus have an association.

### Changes in blood cell numbers and haemoglobin concentrations after irradiation

Next, we confirmed the numbers of WBCs and RBCs and the volumes of haemoglobin (Hb) in blood and haemoglobin in RBCs (mean corpuscular haemoglobin concentration, MCHC) after irradiation. The number of WBCs showed dose-dependent decreases at all four sampling times (Fig. S5a), consistent with a previous report^20^. RBCs and Hb decreased by 30% at day 6 in the 3 Gy irradiation group (P = 0.0502 for RBCs; P = 0.0499 for Hb), suggesting that the i-STrap results were partly affected by the decrease in RBCs (Fig. S5b,c). The MCHC did not change noticeably, suggesting that radiation did not affect haemoglobin in RBCs (Fig. S5d).

### Radiation increases the hydroperoxide level in plasma

**Figure 3:**
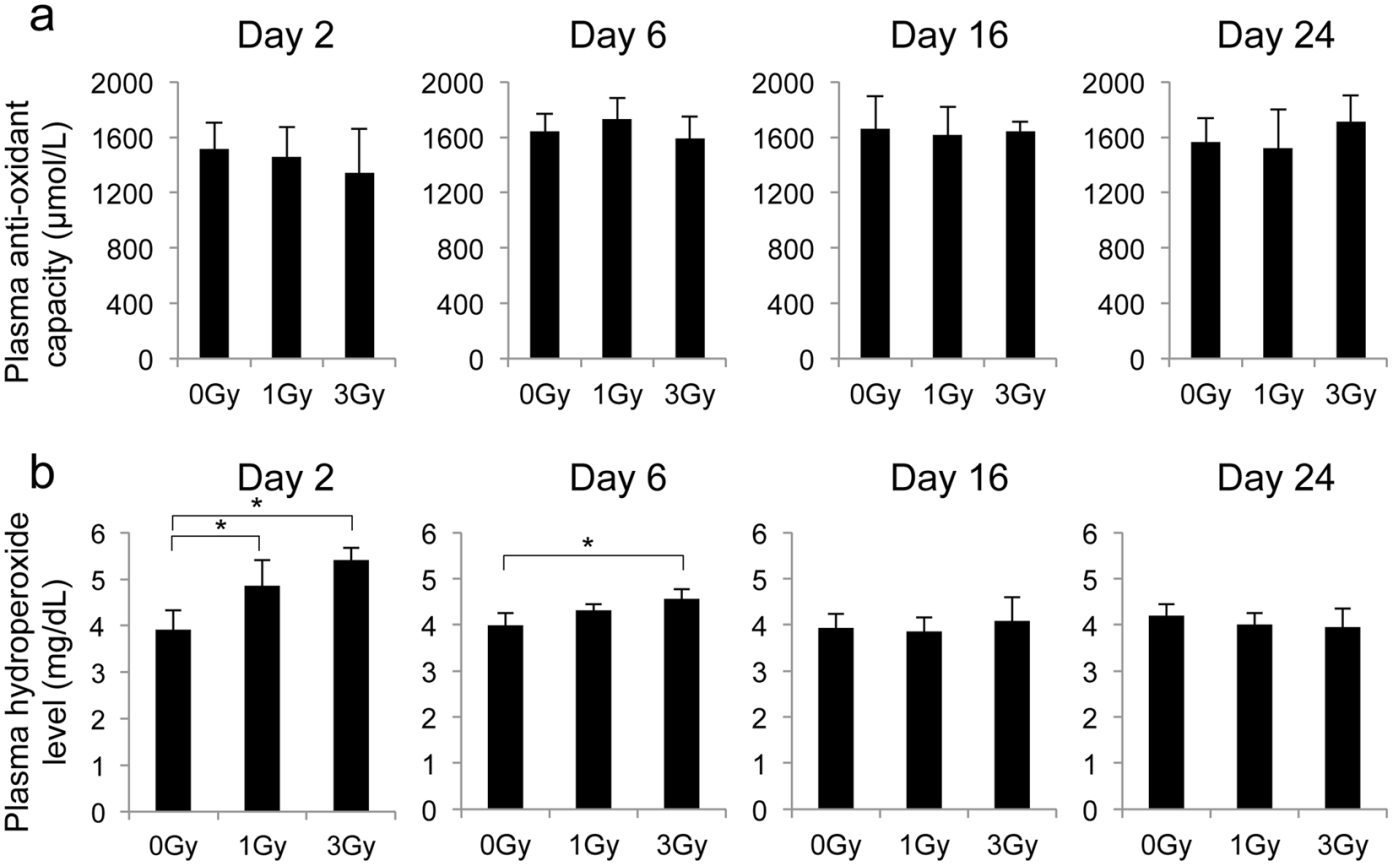
Changes in plasma anti-oxidant capacity and hydroperoxide level after irradiation. (**a**,**b**) Plasma anti-oxidant capacity (**a**) and hydroperoxide level (**b**) were measured by an i-Pack Oxystress Test. All quantitative data are presented as the means ± SD (n = 3–4). *P < 0.05, Welch’s *t*-test.

To investigate whether irradiation affects the redox state of non-cellular components in blood, we measured the plasma hydroperoxide level and anti-oxidant capacity using an i-Pack Oxystress Test. We found that plasma anti-oxidant capacity did not change from 2 to 24 days after irradiation at 1 or 3 Gy (Fig. 3a). Meanwhile, the plasma hydroperoxide level was higher at 2 and 6 days after irradiation than the no-irradiation group, suggesting that radiation increases plasma oxidative stress (Fig. 3b).

## Discussion

ESR spin trapping is a direct and highly credible technique for detecting free radicals *in vitro* and *in vivo*. 2,2-Dimethyl-3,4-dihydro-2H-pyrrole N-oxide (DMPO) is a commonly used spin-trapping agent. However, DMPO can be inconvenient for experiments because its spin adduct stability and half-life are insufficient^6^. In the present study, we chose to use the novel spin-trapping agent DPhPMPO because it is approximately five times more sensitive for reactions with lipid-derived radicals than DMPO. In addition, DPhPMPO spin adducts can be stably maintained at −80 °C (data obtained from Dojindo Co., Kumamoto, Japan). Several reports have described relationships between redox state and disease or body conditions, including radiation exposure. However, almost all of these studies analysed non-cellular components (plasma, serum, urine, or saliva)^7,8^. A recent report showed that RBCs are enriched in redox-related metabolites and proposed the RBC redox state as an important marker for health and body conditions^9^. In this study, we examined whether radiation exposure affects whole-blood anti-oxidant capacity using i-STrap, a new ESR spin-trapping technique using DPhPMPO.

We demonstrated that whole-blood anti-oxidant capacity decreased in a highly dose-dependent manner 2 to 24 days after irradiation at 0.5–3 Gy (Fig. 1), suggesting this capacity’s potential use in biodosimetry for large-scale radiological and/or nuclear incidents. We also found that whole-blood anti-oxidant capacity was not decreased 30 min after irradiation, suggesting that radiation-generated ROS and free radicals did not directly affect whole-blood anti-oxidant capacity (Fig. 1). Unexpectedly, the whole-blood anti-oxidant capacity decrease peaked approximately 1 week after irradiation (Fig. 1). Several studies have described cellular oxidative stress being induced a few hours to a few days after acute irradiation through enhanced mitochondrial electron transport chain function and mitochondrial ROS production^21,22^, and these results may be consistent with our findings. A DNA damage sensor gene, ataxia telangiectasia mutated (ATM), was reported to mediate mitochondrial ROS production after irradiation, suggesting that radiation-induced oxidative stress was mediated by the DNA damage response process^23^. However, these observations cannot completely explain our results because the DNA damage response process was almost complete at 24–48 h after irradiation at 1–3 Gy^24^.

GSH is a typical anti-oxidant in mammals. RBCs contain approximately 100 times higher glutathione levels than plasma^17^. RBC glutathione levels are lower in elderly people^25^ and sickle cell disease patients than in healthy adults^17^ and increase 18 hours after ski marathons^26^, suggesting that the RBC glutathione level is an important marker for health, ageing, and body condition. Navarro *et al*.^27^ measured mouse blood glutathione levels until 24 h after irradiation and found that radiation increased GSSG but did not change GSH. They noted the possibility that the reaction of GSH with radiation-induced free radicals increases GSSG levels or GSSG release from different organs (e.g., liver) into the blood until 24 h after irradiation^27^. In this study, radiation decreased the RBC total glutathione and GSH levels and the GSH/GSSG ratio in a dose-dependent manner at day 2 and day 6 (Fig. 2). Our results suggest that the biosynthesis of GSH was inhibited and/or the reduced GSSG reduction ability in RBCs was increased 2 and 6 days after irradiation.

We found that radiation increased the plasma hydroperoxide level in a dose-dependent manner but did not affect plasma anti-oxidant capacity (Fig. 3). These results are partly consistent and partly inconsistent with previous reports on oxidative stress markers in humans and animals. Malekirad *et al*.^28^ reported that radiology staff showed greater plasma lipid peroxidation and total anti-oxidant capacity than a control (non-irradiated) group. Al-Nimer *et al*.^29^ reported that radiology staff showed a higher plasma malondialdehyde (MDA) level than a control (non-irradiated) group. Serhatlioglu *et al*.^30^ reported that radiological department workers showed a lower anti-oxidant enzyme paraoxonase (PON1) level and a higher MDA level than a control (non-irradiated) group. Urushihara *et al*.^8^ reported that cattle within the ex-evacuation zone of the Fukushima Daiichi nuclear plant accident had higher plasma MDA levels than control (non-irradiated) cattle, while their plasma superoxide dismutase (SOD) activity and glutathione peroxidase (GPx) activity were not significantly different. Furthermore, clinically used total body irradiation doses lead to increased levels of markers of lipid peroxidation in patient plasma^31^. Our findings and those in previous studies consistently indicate that radiation increases oxidant (including MDA and hydroperoxide) levels in plasma over a wide dose range (milligrays to grays), suggesting their potential use as biomarkers in biodosimetry. Furthermore, Nomiya *et al*.^32^ found that the hydroperoxide level increased in rat thigh after irradiation, suggesting that the plasma oxidant level can be an effective biomarker for partial body irradiation. We found that radiation did not change the MCHC, suggesting that low levels of GSH allowed an increase in plasma hydroperoxide through haemoglobin-mediated radical production (Fig. S5d).

Considering this evidence together, we suggest that i-STrap and the measurement of RBC glutathione or plasma hydroperoxide levels have potential use in biodosimetry. However, our results leave several open questions. First, our results suggested that whole-blood anti-oxidant capacity was highly dependent on the concentrations of RBCs or their constituents and was barely affected by the constituents of plasma (Figs S2 and S3). However, our BSO experiments showed that the RBC glutathione level did not affect the i-STrap results (Fig. S4). This finding may be because DPhPMPO has a higher reaction rate than GSH and was used at a higher dose (10 mM) than the blood GSH concentration (approximately 1 mM). Further studies should reveal the kinds of metabolites, enzymes, and proteins that directly affect the results of i-STrap and whether their levels decrease after radiation exposure. Second, we found that the whole-blood anti-oxidant capacity decrease peaked approximately 1 week after irradiation (Fig. 1). However, we still cannot fully explain its mechanisms. The DNA damage response, mitochondrial ROS production, inflammation, and other systemic radiation responses may be related to this phenomenon in a complex manner. Third, most of the radiation effects (cancer, cataracts, and circulatory disease) are correlated with oxidative stress. Therefore, we need to investigate whether our observed failure in blood redox balance causes these diseases.

In conclusion, the present study has shown that radiation affects the redox homeostasis of blood. We found that whole-blood anti-oxidant capacity as well as the RBC glutathione level decreased in a dose-dependent manner. These phenomena were associated with an increase in the plasma hydroperoxide level. Collectively, our findings open a new avenue for the development of novel biodosimetry techniques to estimate radiation exposure after radiological and/or nuclear incidents. Furthermore, redox state is considered a marker for complex biological responses to radiation. Thus, redox state may be a novel marker for estimating the risk rates of radiation exposure effects (including cancer and non-cancer diseases) and predicting the efficacy and toxicity of radiation therapy.

## Materials and Methods

### Mice and irradiation

Six-week-old male C57BL/6J mice were obtained from Japan SLC (Shizuoka, Japan). Their diet and drinking water were sterilized by autoclaving. After at least 1 week of acclimation, the mice received total body irradiation with 0, 0.5, 1, 2, or 3 Gy of X-rays (150 kV; 20 mA; filter: 0.2 mm Cu and 0.5 mm Al; MBR-1520R-3; Hitachi Power Solutions, Ibaraki, Japan). Mouse whole blood was collected by 0.5 mm Goldenrod Animal Lancet (MEDIpoint, New York, USA)^33^ puncture of the submandibular vein at 30 min and 1, 2, 4, 6, 9, 16, 24, and 50 days after irradiation.

For evaluation of the characteristics of i-STrap, 4–12 mice were analysed at each point. To analyse whether anti-oxidant treatment affected the results of i-STrap, 3 mice were analysed in each group. For measurement of whole-blood anti-oxidant capacity (i-STrap) after irradiation, 12–14 mice were analysed at each point. For measurement of RBC glutathione levels after irradiation, 6–8 mice were analysed at each point. For BSO treatment experiments, 6 mice were analysed in each group. For measurement of plasma anti-oxidant capacity and hydroperoxide levels after irradiation, 3–4 mice were analysed at each point. For the measurement of changes in WBCs, RBCs, Hb level, and MCHC after irradiation, 5 mice were analysed at each point.

### Measurement of whole-blood anti-oxidant capacity (i-STrap)

Whole-blood anti-oxidant capacity was measured using a novel ESR spin-trapping technique termed i-STrap (Dojindo, Kumamoto, Japan), according to the manufacturer’s protocol. Whole blood was collected into heparin-containing tubes. Next, 20 μL of DPhPMPO solution (final concentration: 10 mM) (Fig. S1a), 100 μL of saline, and 100 μL of whole blood were mixed with a VORTEX-GENIE 2 Mixer (M&S Instruments, Osaka, Japan), and 20 μL of tBuOOH (final concentration of 10 mM) was then added. After the samples were incubated at room temperature for 30 min, 1 mL of chloroform/methanol (2:1) solution (Wako Pure Chemical Industries, Osaka, Japan) was added. The samples were then mixed with the VORTEX-GENIE 2 Mixer at room temperature for 10 min to extract spin adducts in the chloroform/methanol (organic) layer (Fig. S1b). The samples were centrifuged at 8000 × *g* and 4 °C for 10 min, and the organic layer was collected into a new tube and stored at −80 °C until ESR measurement.

For ESR measurement, the samples were warmed to room temperature, and 160 μL of each sample was drawn into a quartz flat cell (RST-LC09F; Flashpoint, Tokyo, Japan). The samples were measured by X-band ESR spectroscopy (JES-TE200; JEOL, Tokyo, Japan). The ESR conditions were as follows: microwave frequency, 9.422 GHz; microwave power, 2 mW; field centre, 332.0 mT; sweep width, 0.3 mT; sweep time, 4 min; time constant, 0.3 s. Signal of DPhPMPO spin adduct intensity was corrected by marker (manganese oxide; Mn) intensity.

In these experiments (Fig. S1c), tBuOOH reacted with haemoglobin in blood to produce tert-butyl, tert-butyloxyl, and tert-butylperoxyl radicals (Fenton reaction). These radicals competitively reacted with anti-oxidants in blood or DPhPMPO. When the blood contained fewer anti-oxidants (low blood anti-oxidant capacity), more radicals were trapped by DPhPMPO, and a much higher ESR signal was produced.

### Anti-oxidants

Ascorbic acid (Kobayashi Pharmaceutical, Osaka, Japan) and NAC (LKT Laboratories, Minnesota, USA) were dissolved in saline. Trolox (Tokyo Chemical Industry, Tokyo, Japan) was dissolved in DMSO (Wako Pure Chemical Industries).

### BSO treatment

To decrease glutathione levels using a pharmacological method, 10 mM BSO was added to the drinking water, which was administered to the mice for 7 days^18^.

### Measurement of RBC glutathione levels

Whole blood was collected into heparin-containing tubes and centrifuged at 3000 × *g* and 4 °C for 10 min to separate plasma and RBCs. After centrifugation, 100 μL of RBCs was haemolysed with 400 μL of 5% 5-sulfosalicylic acid solution (Wako Pure Chemical Industries), and the samples were centrifuged at 8000 × *g* for 10 min to remove proteins. The supernatants were collected and assayed for glutathione using a GSSG/GSH Quantification Kit (Dojindo) according to the manufacturer’s protocol (Please see: http://www.dojindo.com/store/p/824-GSSG/GSH-Quantification-Kit.html). The absorbance at 412 nm was measured using a Varioskan LUX plate reader (Thermo Fisher Scientific, Kanagawa, Japan). To quantify GSSG and GSH individually, GSH was masked with masking solution before quantification, and GSH was calculated on the basis of the formula GSH = total glutathione − GSSG × 2.

### Measurement of plasma anti-oxidant capacity and hydroperoxide level

Whole blood was collected into heparin-containing tubes and centrifuged at 3000 × *g* and 4 °C for 10 min to separate plasma and RBCs. Plasma anti-oxidant capacity and hydroperoxide level were analysed using an i-Pack Oxystress Test (ARKRAY, Kyoto, Japan)^34^. Absorbance was measured using a Spotchem IM (ARKRAY). Briefly, the anti-oxidant capacity was evaluated by measuring the ability of the sample to reduce ferric (Fe^3+^) ions to ferrous (Fe^2+^) ions. The hydroperoxide level was evaluated by measuring the alkoxy radical (RO•) or peroxy radical (ROO•) levels after the addition of ferric and ferrous ions.

### Complete blood counts

Orbital blood was collected into heparin-containing tubes. The samples were analysed using a pocH®-100*iV* instrument (Sysmex, Hyogo, Japan).

### Statistical analysis

The mean and standard deviation (SD) were calculated for each data point. Welch’s *t*-test was used to analyse the statistical significance of differences between groups. Pearson’s correlation coefficient test was used to analyse the significance of correlation coefficients. Values of P < 0.05 were considered to indicate statistical significance for both tests.

### Ethical considerations

All animal experiments were performed in accordance with the Animal Care Guidelines of the University of Occupational and Environmental Health, Japan (UOEH.J.). The animal husbandry procedures and animal experiments were consistent with the Regulations on Animal Experimentation of the UOEH.J. and were approved by the Animal Experiment Committee of the UOEH.J. (Permit Number: AE15-009). Mice were euthanized after experiments by the administration of CO_2_.

### Data availability

The data that support the findings of this study are available from the corresponding author upon reasonable request.

## Electronic supplementary material

Supplementary information
