# Supplementary material for: Dose-dependent decrease in anti-oxidant capacity of whole blood after irradiation: A novel potential marker for biodosimetry
Source: Sci Rep. 2018 May 9;8:7425. doi: 10.1038/s41598-018-25650-y (PMC5943295; doi:10.1038/s41598-018-25650-y)
Supplement: Supplementary file 1 — Supplementary information [file 41598_2018_25650_MOESM1_ESM.pdf]

Dose-dependent decrease in anti-oxidant capacity of whole blood after irradiation: A novel potential marker for biodosimetry

Lue Sun[1,2], Yohei Inaba[3,4], Keizo Sato[5], Aki Hirayama[6], Koji Tsuboi[1], Ryuji Okazaki[2], Koichi Chida[3,4] and Takashi Moritake[2,*]

[1]Department of Radiation Biology, Faculty of Medicine, University of Tsukuba, 1-1-1 Tennodai, Tsukuba, Ibaraki 305-0006, Japan

[2]Department of Radiological Health Science, Institute of Industrial Ecological Sciences, University of Occupational and Environmental Health, Japan, 1-1 Iseigaoka, Yahatanishi-ku, Kitakyushu, Fukuoka 807-8555, Japan

[3]Course of Radiological Technology, Health Sciences, Tohoku University Graduate School of Medicine, 2-1 Seiryo, Aoba, Sendai, Miyagi 980-8575, Japan

[4]Department of Radiation Disaster Medicine, International Research Institute of Disaster Science, Tohoku University, Aramaki Aza-Aoba 468-1, Aoba-ku, Sendai, 980-0845, Japan

[5]School of Pharmacy, Kyushu University of Health and Welfare, 1714-1 Yoshino Nobeoka, Miyazaki 882-8508, Japan

[6]Center for Integrative Medicine, Tsukuba University of Technology, Kasuga 4-12-7, Tsukuba 305-8521, Japan

[*] moritake@med.uoeh-u.ac.jp

Figure S1

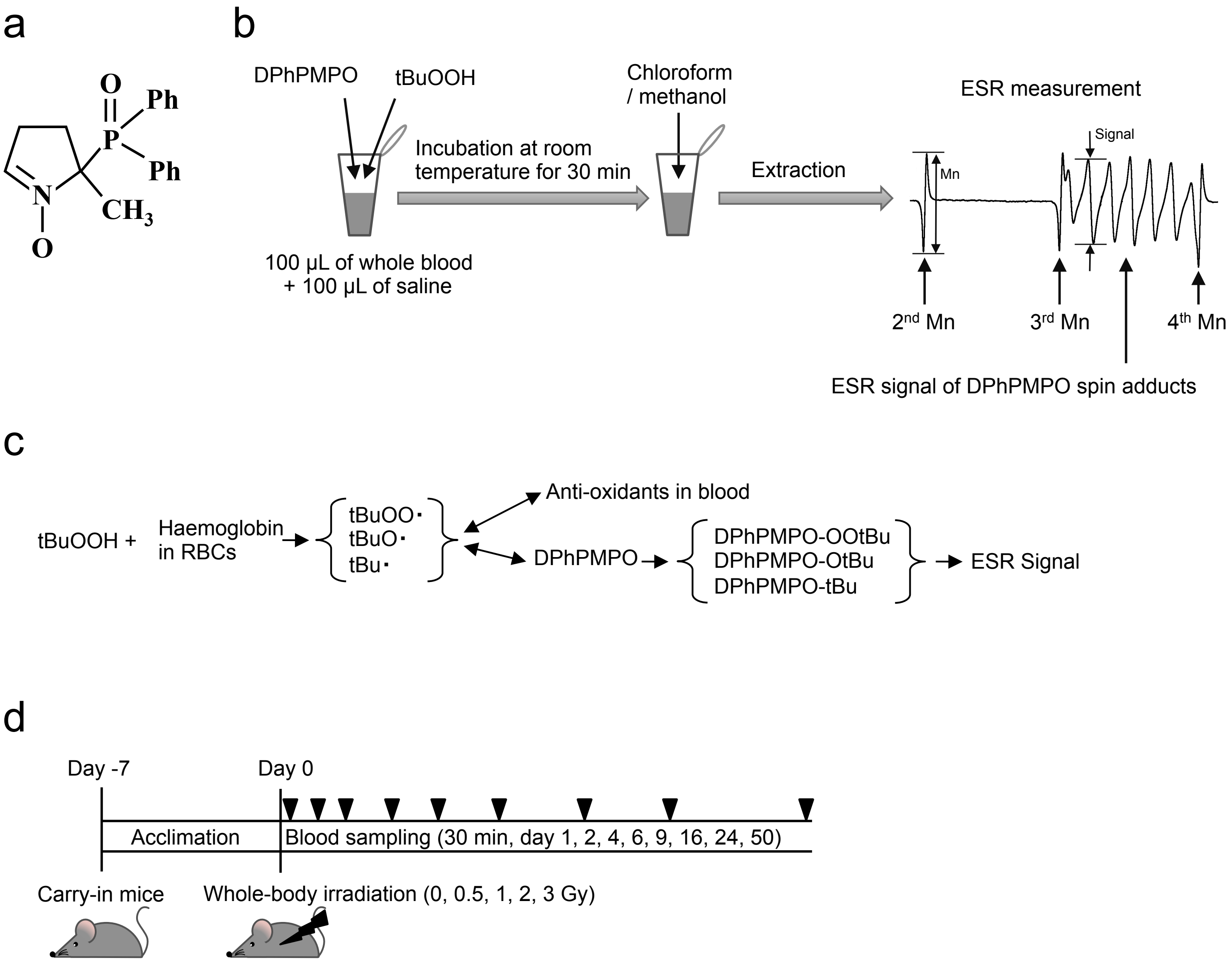

Figure S1. Schema of experiments. (a) Chemical structure of DPhPMPO. (b) Schema of i-STrap. (c) Radical generation and scavenging during i-STrap. (d) Protocol for mouse experiments.

Figure S2

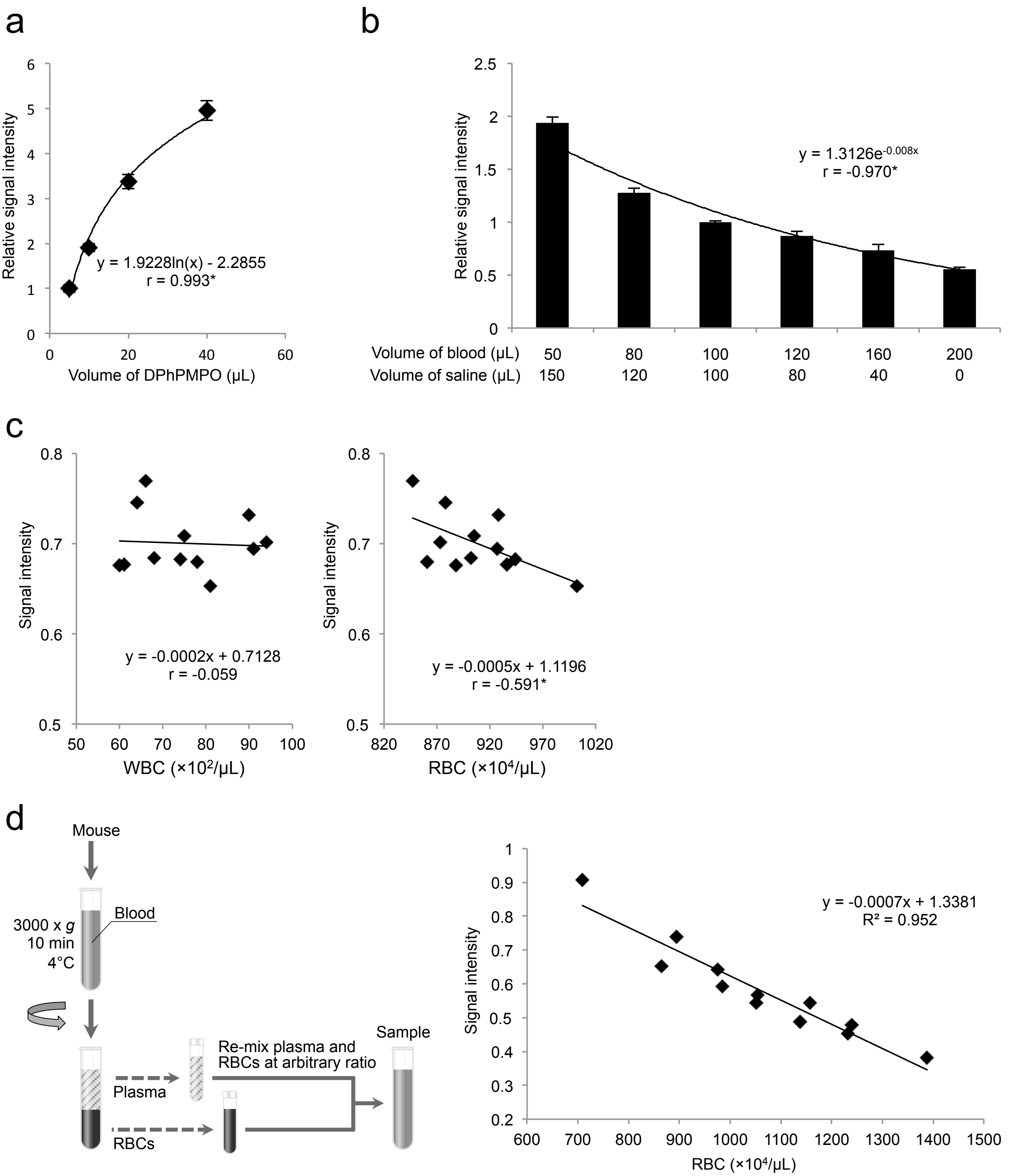

Figure S2. Characterisation of i-STrap. (a, b) Changes in signal intensity with changes in DPhPMPO dose (a) and blood/saline ratio (b). (c) Correlations between signal intensity and numbers of WBCs and RBCs in 12 healthy (non-irradiated) mice. (d) Correlation between signal intensity and number of RBCs in re-mixed blood samples. Quantitative data in (a) and (b) are means ± SD (n = 3). *P < 0.05, Pearson's correlation coefficient test.

Figure S3

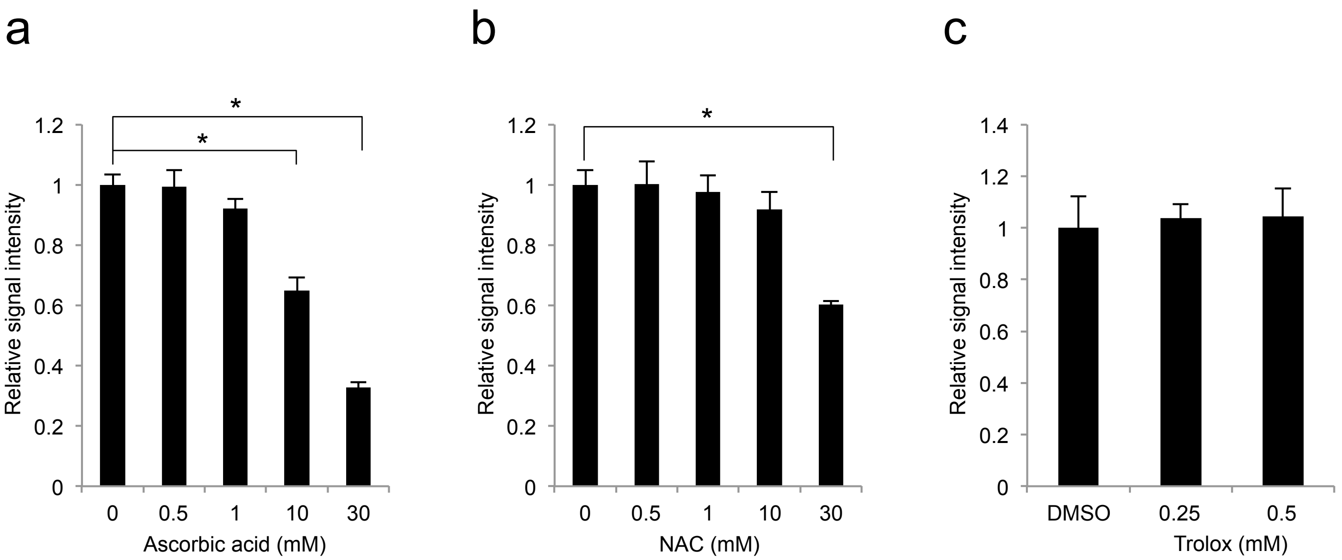

Figure S3. Changes in i-STrap signal intensity with changes in anti-oxidant dose. (a–c) Data for ascorbic acid (a), NAC (b), and Trolox (c). Quantitative data are means ± SD (n = 3). *P < 0.05, Welch's *t*-test.

Figure S4

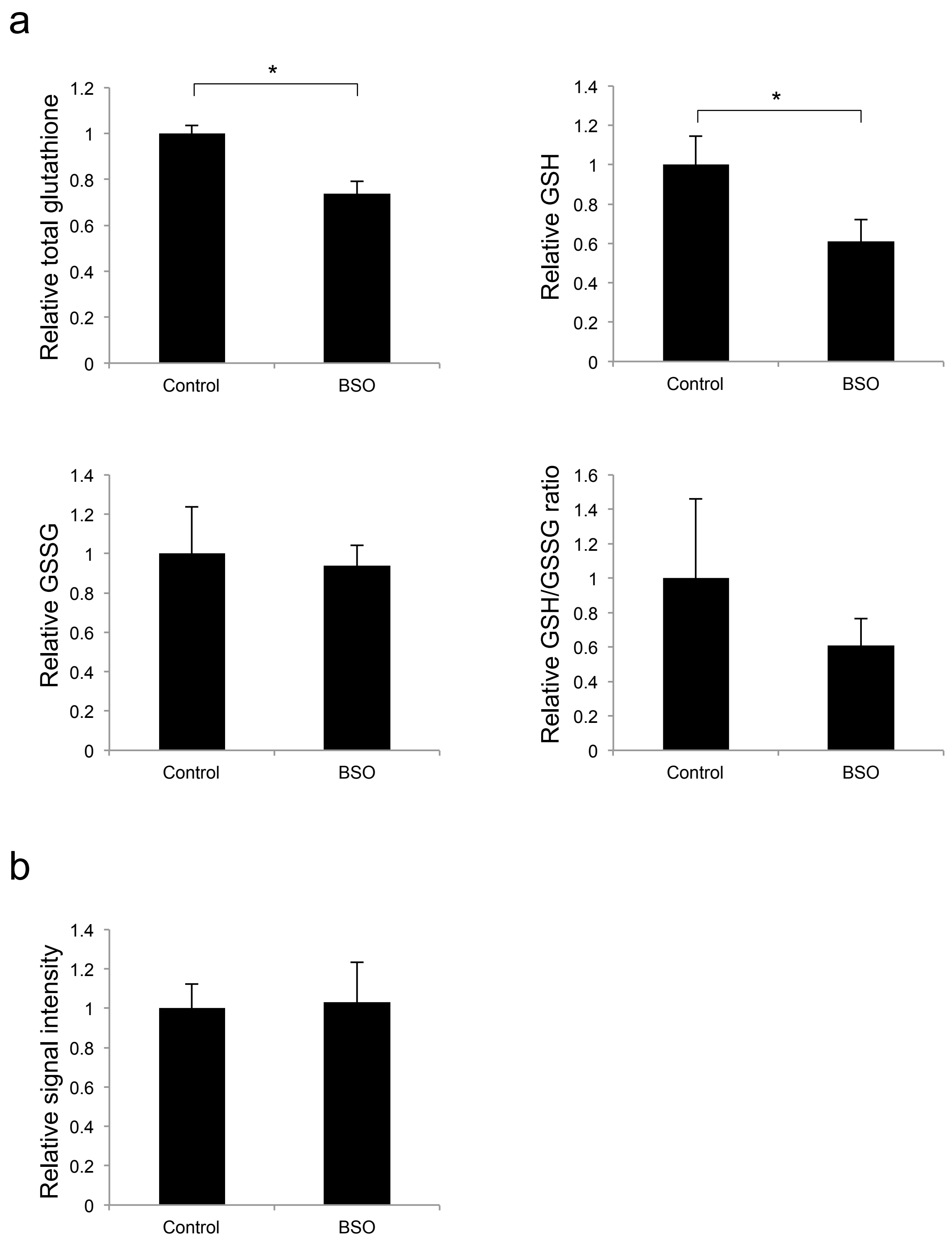

Figure S4. Changes in RBC glutathione level and whole-blood anti-oxidant capacity after BSO treatment. BSO (10 mM) was added to drinking water and administered to mice for 7 days. (a) RBC total glutathione, GSH, GSSG, and GSH/GSSG ratio after BSO treatment. (b) Whole-blood anti-oxidant capacity after BSO treatment measured using i-STrap. Quantitative data are means ± SD (n = 6). *$P < 0.05$, Welch's *t*-test.

Figure S5

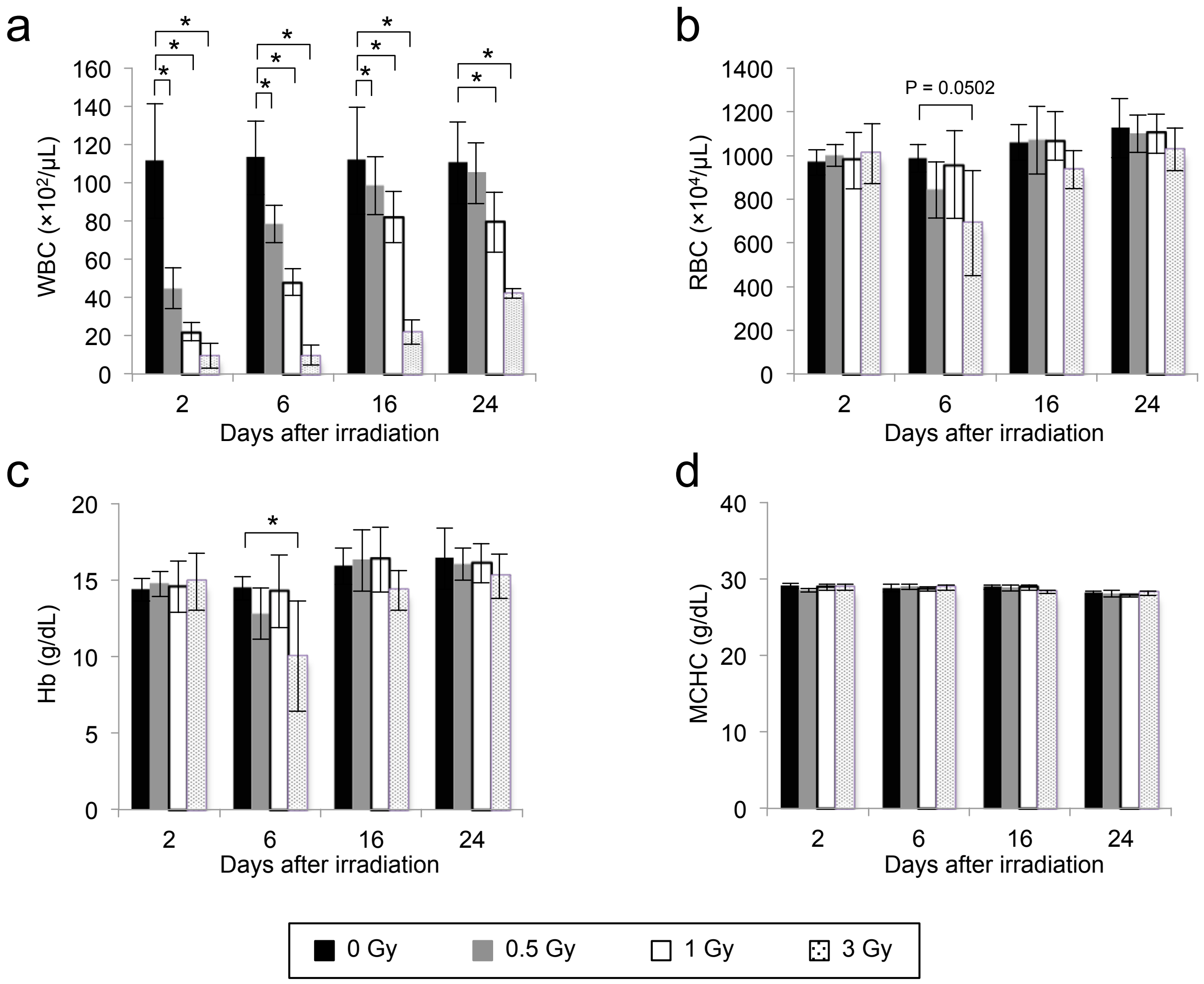

Figure S5. Changes in WBCs, RBCs, Hb, and MCHC after irradiation. (a–d) Changes in numbers of WBCs (a), numbers of RBCs (b), Hb (c), and MCHC (d). All quantitative data are means ± SD (n = 5). *$P < 0.05$, Welch's *t*-test.
